# Using Lorenz Curves to Measure Racial Inequities in COVID-19 Testing

**DOI:** 10.1001/jamanetworkopen.2020.32696

**Published:** 2021-01-08

**Authors:** Aaloke Mody, Kristin Pfeifauf, Elvin H. Geng

**Affiliations:** 1 Division of Infectious Diseases, Department of Medicine, Washington University School of Medicine in St Louis, St Louis, Missouri

## Abstract

This cross-sectional study uses Lorenz curves as a metric for quantifying racial inequities in coronavirus disease 2019 (COVID-19) testing.

## Introduction

Racial disparities have been widely documented during the coronavirus disease 2019 (COVID-19) pandemic, but there has been limited focus on equitable allocation of the pandemic’s most critical but limited resource: COVID-19 testing. Equitable testing is paramount to a successful COVID-19 response and is essential for early case detection, self-isolation, and overall prevention of onward transmission.^1,2,3^ We adapted a well-established tool for measuring inequity from economics—the Lorenz curve^4^—to put forth a metric for quantifying COVID-19 related inequities.

## Methods

This cross-sectional study was reviewed by the Washington University School of Medicine institutional review board and was deemed to be non–human subjects research because data were deidentified; thus, informed consent was not sought. This study follows the Strengthening the Reporting of Observational Studies in Epidemiology (STROBE) reporting guideline for cross-sectional studies.

We examined testing disparities across 7 counties in the St Louis region (total population, 2 149 222 individuals) using data on all COVID-19 tests conducted in Missouri from the State Public Health Department, data on all COVID-19 hospitalizations from the 3 major hospital networks in the region (ie, BJC HealthCare, Mercy, and SSM), and zip code–level data from the 2018 American Community Surveys. Testing and hospitalization data were at the individual level and included information on age, race, zip code, and dates.

We generated modified Lorenz curves to assess disparities in COVID-19 testing relative to disease burden on the basis of the premise that equitable testing is defined by the balance between the number of tests and the actual disease burden, rather than simply achieving an equal number of tests per person (ie, equal testing).^1,2^ We used COVID-19 hospitalizations as the metric for disease burden and generated curves by plotting the cumulative proportion of hospitalizations on the x-axis and the cumulative proportion of tests on the y-axis. We used zip codes as the unit of analysis and color-coded them by their overall racial makeup. With equitable distribution, curves follow a straight, 45° line but become more convex with increasing inequity.^4^ We estimated the Gini coefficient (0 indicating perfect equity and 1 indicating perfect inequity) and Hoover index (the percentage of testing reallocation needed to achieve equity). Additionally, we generated bubble plots of testing rates per hospitalization in Black vs White residents of the same zip code. We performed multiple imputation (50 imputations) to address missingness for race (59.2%) and zip code (15.4%) in the COVID-19 testing data, both of which were highly associated with testing laboratory, date, and result. *P* < .05 from 2-sided Kruskal-Wallis tests was considered significant. All analyses were conducted using Stata MP statistical software version 16.1 (StataCorp) and R statistical software version 3.2.4 (R Project for Statistical Computing).

## Results

Between March 14, 2020, and August 10, 2020, there were 404 904 COVID-19 tests and 4059 hospitalizations across 7 counties in the St Louis region. Lorenz curves depict that only 89 341 tests (22.9%) were conducted in the 23 zip codes accounting for 50% of hospitalizations; 17 of these zip codes were more than 50% Black. In contrast, 218 057 tests (52.9%) were conducted in the 86 zip codes accounting for only 25% of hospitalizations; none of these zip codes was greater than 50% Black (Figure, panel A and Table). The Gini coefficient was 0.401 and the Hoover index was 0.304. Within the same zip code, Black residents had consistently lower rates of tests per hospitalization compared with White residents (Figure, panel B).

**Figure.**
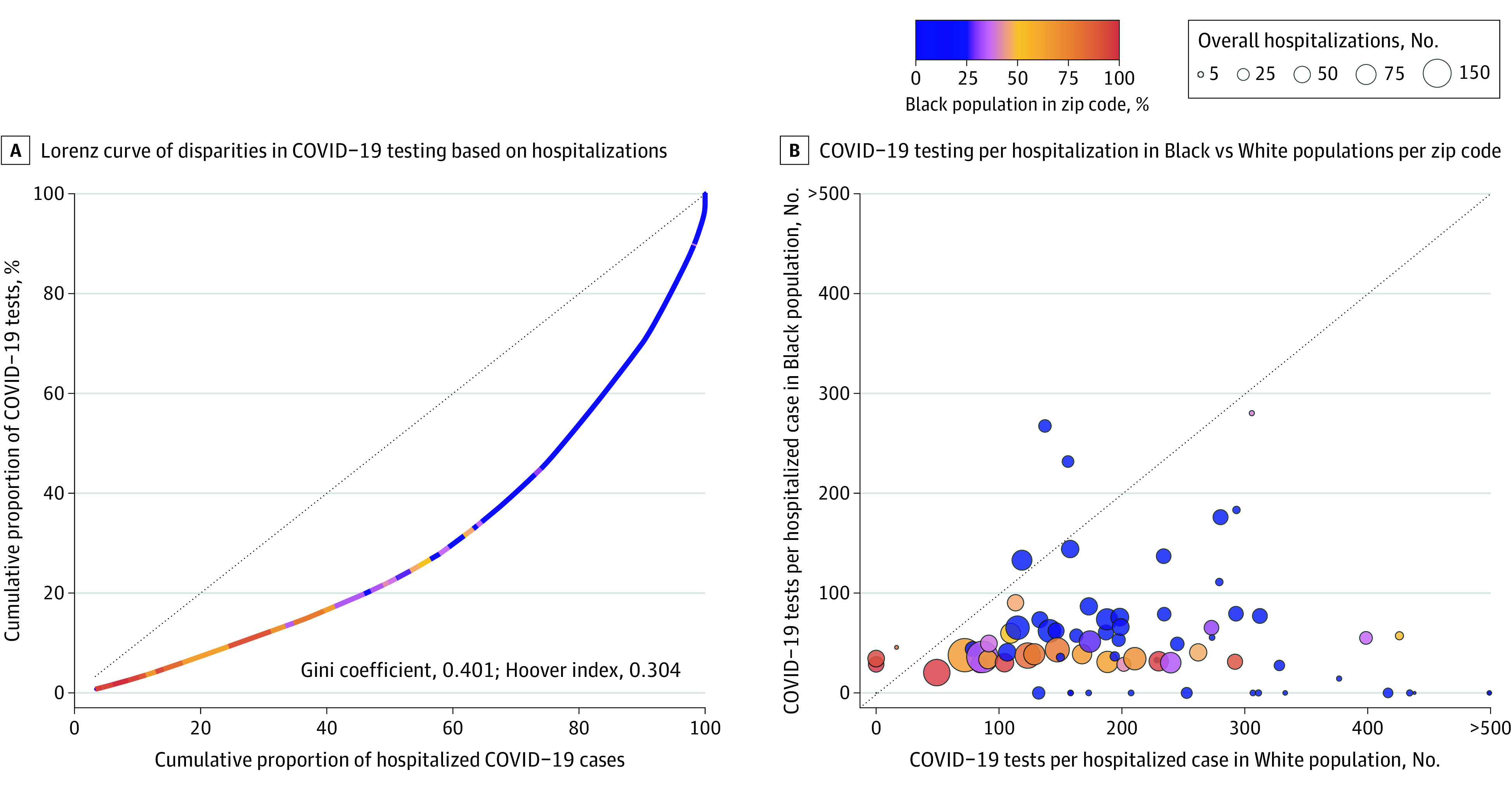
Disparities in Coronavirus Disease 2019 (COVID-19) Testing Relative to Hospitalizations in 7 Counties in the St Louis Region, March 14, 2020, to August 10, 2020 A, Modified Lorenz curve examining disparities in COVID-19 testing relative to the disease burden. The units of analysis are zip codes and they are color-coded by their overall racial makeup. The dashed line represents equitable distribution where 50% of testing would be conducting in zip codes accounting for 50% of hospitalizations. B, Testing rates in Black vs White residents of the same zip code. Each bubble represents a zip code. Bubbles are color-coded by the racial makeup of the zip code and sized by the absolute number of COVID-19 hospitalizations. The dashed line represents equal rates of testing per hospitalization between Black and White residents, with zip codes falling below it having increased testing rates in White residents and zip codes falling above it having increased testing rates in Black residents.

**Table.** Zip Code Characteristics by Quartiles of the Lorenz Curve

Characteristic	Quartile, median (IQR)^a^				*P* value
	Lowest (n = 13)	Second (n = 10)	Third (n = 26)	Highest (n = 86)	
Zip code population, No. of individuals	11 371 (8691-17 879)	19 392 (15 234-21 869)	18 826 (8978-28 909)	6959 (1913-20 550)	.01
Black, %	89.0 (66.4-93.9)	53.3 (34.0-77.6)	10.8 (0.6-36.2)	1.2 (0.1-2.9)	<.001
Male, %	46.5 (44.4-48.1)	47.0 (46.3-48.0)	48.3 (48.0-50.3)	49.6 (48.3-52.1)	<.001
Age, y	38.1 (34.1-40.6)	34.1 (32.6-35.7)	37.7 (35.5-40.3)	40.8 (37.9-45.4)	<.001
Mean household size	3.2 (3.2-3.4)	3.2 (3.0-3.3)	3.0 (2.9-3.1)	3.0 (2.9-3.1)	<.001
Annual household income, $	28 180 (23 911-39 639)	42 710 (38 682-46 009)	58 122 (52 145-64 097)	63 020 (51 267-79 930)	<.001
Below poverty line, %	27.1 (16.7-33.3)	13.4 (7.8-16.5)	7.8 (5.7-15.0)	5.2 (2.7-9.1)	<.001
Employed in health care, %	30.1 (28.7-32.9)	24.0 (22.0-30.8)	22.8 (19.2-25.4)	21.5 (16.7-25.0)	<.001
Employed in service industries, %	34.9 (26.0-36.1)	20.4 (19.9-22.7)	16.8 (13.8-19.9)	14.7 (11.3-18.0)	<.001
Who commute via public transport, %	14.7 (6.8-21.2)	7.1 (3.1-9.7)	1.2 (0.2-4.6)	0.1 (0.0-0.4)	<.001
Work from home, %	3.0 (2.1-4.7)	3.6 (2.3-4.3)	4.0 (2.9-6.1)	4.7 (2.8-7.1)	.07
COVID-19 tests per 100 000 residents, No.	21 348 (19 144-23 109)	22 637 (19 614-25 263)	17 705 (15 880-20 709)	14 933 (12 500-18 637)	<.001
Cases per 100 000 residents, No.					
Diagnosed	2349 (2025-2719)	2257 (2064-2609)	1597 (1110-1842)	903 (380-1366)	<.001
Hospitalized	560 (495-592)	410 (382-501)	199 (145-232)	55 (0-86)	<.001
Deaths per 100 000 residents, No.	84 (61-131)	115 (73-156)	31 (0-67)	0 (0-26)	<.001
COVID-19 tests per case, No.					
Diagnosed	9.1 (7.4-9.9)	9.6 (8.8-10.4)	12.2 (10.5-15.2)	16.3 (12.9-24.9)	<.001
Hospitalized	38 (33-43)	53 (46-59)	104 (84-115)	232 (166-324)	<.001
COVID-19 tests per death, No.	202 (92-278)	203 (149-304)	618 (317-2387)	3236 (570-30 512)	<.001

Abbreviations: COVID-19, coronavirus disease 2019; IQR, interquartile range.

^a^ Each quartile corresponds to successive segments of the Lorenz curve so that each quartile contains sufficient consecutive zip codes to account for 25% of hospitalizations in the St Louis region. Lorenz curve–based quartiles were generated by first sorting zip codes by their ratio of COVID-19 tests to hospitalizations and splitting them such that each quartile accounted for 25% of the overall hospitalizations. Thus, the first quartile represents zip codes on the leftmost side of the curve (ie, have the lowest ratio of COVID-19 tests to hospitalizations) and the last quartile represents the zip codes on the rightmost side of the curve (ie, have the highest ratio of COVID-19 tests to hospitalizations). *P* values were generated with 2-sided Kruskal-Wallis tests to assess differences between quartiles.

## Discussion

Our analysis demonstrates the inequitable allocation of COVID-19 testing relative to the disease burden between Black and White communities in the St Louis region, both across and within zip codes. A key component of an equitable testing strategy is that testing needs to be scaled up relative to the disease burden to ensure that not just the most symptomatic cases are identified in an area.^1,2,3^ Lorenz curves provide straightforward metrics that can also be easily tracked over time to quantify these disparities. Black communities have been disproportionately affected by COVID-19, and these methods demonstrate how inequitable and inadequate testing scale-up may be an important factor associated with the disparities. The underlying reasons for undertesting in particular communities are likely several fold and include existing disparities in the health care infrastructure, access to health care, and mistrust of historically discriminatory health care system, all of which are manifestations of structural racism in our current health care system.^5,6^ Addressing these inequities likely requires proactive public health responses, such as targeted use of high-volume, saliva-based tests and community-based testing campaigns.^1,2^ Study limitations include missingness in race and zip code from the programmatic COVID-19 testing data.
